# Lifestyle Modification in Prediabetes and Diabetes: A Large Population Analysis

**DOI:** 10.3390/nu17081333

**Published:** 2025-04-11

**Authors:** Michael L. Dansinger, Joi A. Gleason, Julia Maddalena, Bela F. Asztalos, Margaret R. Diffenderfer

**Affiliations:** 1 Boston Heart Diagnostics Corporation, 200 Crossing Boulevard, Framingham, MA 01702, USA; joigleason@comcast.net (J.A.G.); julia.maddalena@gmail.com (J.M.); bela.asztalos7@gmail.com (B.F.A.); 2 Jean Mayer USDA Human Nutrition Research Center on Aging, Tufts University, Boston, MA 02111, USA

**Keywords:** diabetes, prediabetes, HOMA_IR_, fasting glucose, insulin, ASCVD risk, lifestyle

## Abstract

**Background/Aims**: Diabetes mellitus is a major cause of atherosclerotic cardiovascular disease (ASCVD). We examined a large population and tested the efficacy of a voluntary lifestyle program in prediabetic and diabetic subjects. **Methods**: Of 133,764 subjects, 56.3% were healthy, 36.2% were prediabetic, and 7.5% were diabetic. Fasting serum measurements of glucose, insulin, adiponectin, glycosylated hemoglobin (HbA1c), high-sensitivity C-reactive protein (hs-CRP), glycated serum protein (GSP), fibrinogen, myeloperoxidase (MPO), lipoprotein-associated phospholipase A_2_ (LpPLA_2_), as well as standard lipids, direct low-density lipoprotein cholesterol (LDL-C), and small dense LDL-C (sdLDL-C) were performed using standard automated assays. Follow-up sampling at 6–12 months occurred in 20.1% of the prediabetic and 22.2% of the diabetic subjects; of these, 12.2% of the prediabetic and 9.7% of the diabetic subjects participated in a voluntary, real-world, digital dietitian-directed lifestyle-modification program with a 10-year diabetes risk being calculated using a biochemical model (Framingham). **Results**: Prediabetic and diabetic subjects had significantly elevated triglycerides, sdLDL-C, and hs-CRP and decreased HDL-C. They were insulin resistant as compared to healthy subjects, but only diabetics had significant reductions in insulin production. Lifestyle modification significantly reduced diabetes risk by 45.6% in prediabetics and significantly increased (2.4-fold) the percentage of diabetics that were in remission at follow-up (8.2% versus 3.4%) with increased weight loss (6.5 versus 2.0 pounds). Lifestyle intervention resulted in significant favorable effects on many metabolic markers. **Conclusions**: The measurement of fasting glucose and insulin is essential for the detection of decreased insulin production in diabetics. A digital lifestyle program can have favorable effects on ASCVD risk factors and diabetic status.

## 1. Introduction

Current criteria for the diagnosis of diabetes mellitus in the United States are having a fasting plasma glucose ≥ 126 mg/dL (7.0 mmol/L) and/or a glycosylated hemoglobin (HbA1c) value ≥ 6.5% according to the American Diabetes Association [1]. Subjects with diabetes are known to be at significantly higher risk of developing atherosclerotic cardiovascular disease (ASCVD), kidney disease, diabetic retinopathy, and neuropathy [1,2]. Prediabetes has been defined as having a fasting plasma glucose of 100–124 mg/dL and/or having a HbA1c value of 5.7–6.4%; about one third of the middle-aged and elderly United States population have this condition [1]. Having prediabetes, being overweight or obese, being >45 years of age, having a parent or sibling with type 2 diabetes, being physically inactive (<3 times a week), having gestational diabetes (diabetes during pregnancy) or giving birth to a baby who weighed more than 9 pounds (4.1 kg), and/or race or ethnic background have all been associated with an increased diabetes risk according to the American Diabetes Association [1].

It is recommended that patients with prediabetes be placed on an intensive behavioral lifestyle intervention program, modeled on the Diabetes Prevention Program, to achieve and maintain 7% loss of initial body weight and increase moderate-intensity physical activity (such as brisk walking) to at least 150 min/week [1]. Intensive lifestyle intervention using exercise (mainly walking for 30 min/day or more) and caloric control (restriction of high fat, high sugar desserts) has been shown to reduce the risk of developing diabetes by more than 50% in subjects with prediabetes [1,2,3,4,5,6]. In a review of randomized trials in which 5238 prediabetics were enrolled for 2–6 years, about 15% developed diabetes in the treatment group versus about 26% in the control group, representing a 42% risk reduction [6]. However, such intensive programs involve multiple in-person sessions and have not been widely adopted in clinical practice.

Therefore, it is important to develop risk prediction tools to identify subjects at high risk for developing diabetes mellitus as well as to test high-risk subjects with an intervention that can easily be implemented. Investigators have previously developed models for predicting diabetes over 5–10 years using a variety of markers, including fasting glucose, body mass index (BMI), high-density lipoprotein cholesterol (HDL-C), parental diabetes history, and triglyceride levels [7,8,9,10,11,12,13]. Noble and colleagues reviewed data from 94 diabetes risk prediction models tested in a large number of individuals [9]. Follow-on studies that applied a risk score as part of an intervention aimed at reducing actual risk in people were sparse.

Recently we have developed a diabetes prediction model based on prospective data from the Framingham Offspring Study [13]. This model predicts the 10-year risk of developing diabetes and uses the following four biochemical parameters based on fasting blood sampling: (1) glucose, (2) glycated serum albumin, (3) adiponectin, and (4) triglycerides. The model has a C statistic of 0.897. As previously stated, lifestyle modification remains the cornerstone of therapy for both ASCVD and diabetes prevention, often using diets restricting calories, animal and trans fats and sugars, and an exercise program, along with medications if necessary [1,2,3,4,5,6]. We have previously developed and tested a lifestyle modification program that has been shown to be effective in promoting weight loss, as well as lowering serum triglyceride levels, reducing insulin resistance, and increasing HDL-C levels [14]. Our goals in this study were (1) to apply our Framingham biochemical diabetes risk model to a large population and (2) to assess the efficacy of a voluntary online lifestyle modification program in reducing ASCVD risk and improving diabetes status.

## 2. Materials and Methods

### 2.1. Human Subjects and Study Design

Studies were carried out as an anonymized, retrospective analysis of a total of 135,929 subjects whose physicians sent serum samples (after blood drawing, allowing samples to clot for no more than 30 min, centrifugation, and aliquoting), shipped on ice packs by over-night courier service, to Boston Heart Diagnostics (Framingham, MA, USA) for ASCVD risk assessment; the subjects also had testing 6–12 months later. Only data from fasting subjects were used. All data were anonymized prior to analysis, and such analyses are exempt from institutional review board review [exemption 4, https://grants.nih.gov/policy/humansubjects.htm (accessed on 9 September 2020), see open education resource website 45 CFR 46.104(d)]. Of these 135,929 subjects, 1.6% were excluded because they were listed as being diabetic and on insulin therapy. Of the remaining 133,774 subjects, based on Boston Heart testing over a 3-year period of time, 56.3% were classified as healthy (fasting glucose < 100 mg/dL), 36.2% were classified as having prediabetes (fasting glucose 100–125 mg/dL), and 7.5% were classified as having diabetes (fasting glucose ≥ 126 mg/dL or receiving diabetes medication, but not insulin) (Table 1).

All prediabetic and diabetic subjects were able to access a free, personalized diet and exercise plan electronically via an online portal, with online tools for tracking diet, exercise, and weight. The plan provided a single counseling session and review with a dietitian, as previously described [14]. It also provided multiple menus with recommended carbohydrate intakes ranging from 30% to 60% (median 45%) and saturated fat intakes ranging from 5% to 10% but no more than 7% for subjects with known ASCVD or low-density lipoprotein cholesterol (LDL-C) ≥ 160 mg/dL. The consumption of unrefined, natural foods such as vegetables, legumes, fruits, low-fat dairy, and whole grains was promoted. Total calorie intake was set to facilitate healthy weight as determined by the waist-to-height ratio. A 30% reduction in calories was recommended for those requiring weight loss. Caloric intake was estimated using the Mifflin–St. Jeor equation. Macronutrient and calorie targets were translated into food servings and personalized 7-day menus that were consistent with the dietary approaches to stop hypertension and with Mediterranean diets [14].

It is important to emphasize that this was not a randomized controlled trial but rather a retrospective analysis of our experience with a voluntary “real-world” lifestyle intervention program. The lifestyle evaluation occurred over a 6–12 month period based on laboratory values. There was no dietary compliance assessment. Only those subjects with no change in diabetic medications were included in the analysis of the efficacy of the lifestyle intervention program.

### 2.2. Laboratory Measurements

**Figure 1:**
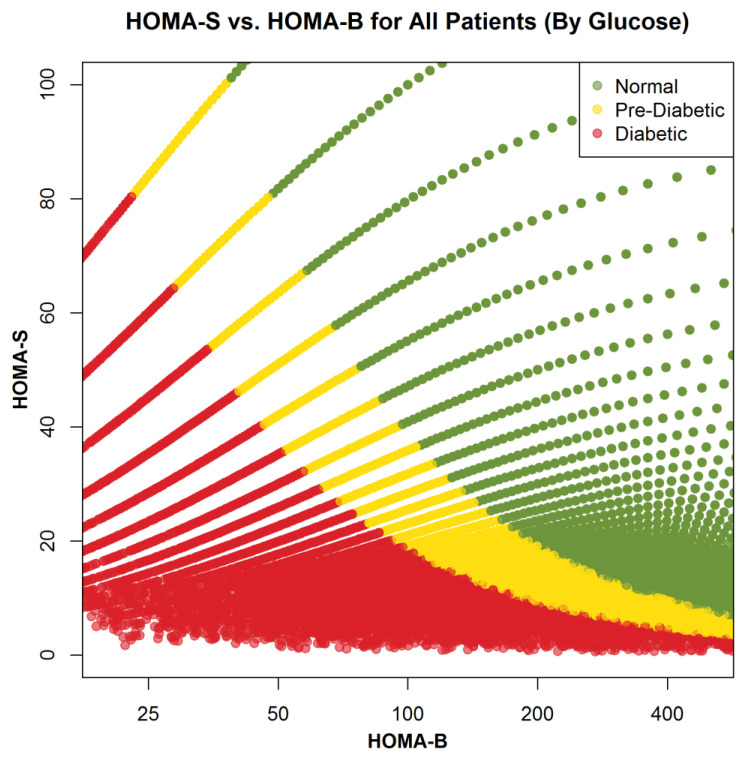
Insulin production and insulin sensitivity in healthy, prediabetic, and diabetic subjects. In this figure we have plotted data for the entire population of 133,764 subjects (56.3% healthy, 36.2% prediabetic, and 7.5% diabetic). Homeostasis assessment model assessment of insulin production, or HOMA_β_, was calculated as equal to [360 × fasting insulin (µU/mL)]/[fasting plasma glucose (mg/dL) − 63] as previously described and plotted on the horizontal axis (22). The homeostasis model of insulin resistance, or HOMA_IR_, was calculated as equal to [fasting insulin (µU/mL)] × [fasting plasma glucose (mg/dL)]/405 as previously described [22]. We then plotted the reciprocal of this value multiplied by 100, or as [(1/HOMA_IR_) × 100], for the same subjects as a measure of insulin sensitivity (HOMA_S_). What can be clearly seen on the graph is that diabetic subjects not infrequently have HOMA_β_ of <60 (the 25th percentile value in healthy subjects), as well as decreased insulin sensitivity as compared to healthy and prediabetic subjects, with clear lines of demarcation between diabetic, prediabetic, and healthy subjects.

The fasting serum or red blood cell specimens sent to Boston Heart Diagnostics were measured for glucose, insulin, C-peptide, high sensitivity C-reactive protein (hs-CRP), HbA1c, direct LDL-C, direct small dense low-density lipoprotein cholesterol (sdLDL-C), and HDL-C using assays obtained from Roche Diagnostics (Indianapolis, IN, USA) and for adiponectin and glycated serum protein (GSP) using assays obtained from Diazyme Laboratories (Poway, CA, USA). All assays were performed on Roche COBAS analyzers and had within- and between-run CVs of <4.0%, as previously described [15,16,17,18,19,20,21]. With regard to the measurement of serum glucose, we have compared the values obtained with those obtained using plasma and have found that the results were virtually identical (<2.0% difference), consistent with the Roche package insert for this assay. For all populations, homeostasis model of insulin resistance (HOMA_IR_) values were calculated as [fasting insulin (µU/mL) × fasting plasma glucose (mg/dL)]/405, while homeostasis model of insulin production (HOMA_β_) values were calculated as [360 × fasting insulin (µU/mL)]/[fasting plasma glucose (mg/dL) − 63], as previously described [22]. As shown in Figure 1, we plotted the homeostasis model of insulin sensitivity (HOMA_s_) values, calculated as [(1/HOMA_IR_) × 100], versus HOMA_β_ values in all subjects. We also plotted the reciprocal of this value multiplied by 100, or as [(1/HOMA_IR_) × 100], for the same subjects as a measure of insulin sensitivity (HOMA_S_) [Figure 1].

### 2.3. Statistical Analysis

For all populations, biochemical variables are expressed as median values with the interquartile range (25–75th percentile). Variables that were not normally distributed, including adiponectin, glycated albumin, hs-CRP, insulin, and triglycerides, were log-transformed before statistical analysis. Categorical variables are provided by percentage.

All variables between subjects that were healthy, prediabetic, or diabetic were compared using the non-parametric Kruskal–Wallis test. To predict diabetes risk in healthy subjects and prediabetic subjects in the Boston Heart Diagnostics population, we used a biochemical model that included fasting glucose, fasting triglycerides, adiponectin, and glycated serum protein with a C statistic of 0.897, as described [13].

## 3. Results

### 3.1. Comparison of Healthy, Prediabetic, and Diabetic Subjects—Age and Weight

As shown in Table 1, the percentage of women was decreased in the prediabetic group (−26.4%) and in the diabetic group (−35.4%), compared to the healthy group, indicating a sex effect. Prediabetic and diabetic men (+11.5%; +15.4%, respectively) and women (+15.5%; +15.4%, respectively) were significantly older than healthy subjects, indicating a significant age effect. As expected, BMI values were significantly higher in prediabetic and diabetic men (+7.1%; +10.7%) and women (+15.4%; +30.8%), indicating a strong obesity effect as compared to healthy subjects. Similar differences were seen for body weight.

### 3.2. Comparison of Healthy, Prediabetic, and Diabetic Subjects—Glucose Homeostasis

As shown in Table 1 and Table 2, HbA1c values in prediabetic and diabetic men (+3.6%; +30.9%) and women (+5.6%; +33.3%) were significantly higher than in healthy subjects. Fasting serum glucose values in prediabetic and diabetic subjects by selection were significantly higher in men (+15.2%; +68.5%) and women (+16.7%; +70.0%) as compared to healthy subjects. GSP in prediabetic and diabetic subjects was significantly higher in men (+4.0%; +52.5%) and women (+1.0%; +43.9%) than in controls, especially in the diabetic group. Fasting insulin levels were significantly higher in prediabetic and diabetic subjects in men (+44.4%; +88.9%) and women (+62.5%; +125.5%) than in controls. Fasting C-peptide values in prediabetic and diabetic subjects were similarly significantly higher in men (+39.1%; +65.2%) and women (+60.0%; +90.1%) than in controls.

The greatest difference seen, however, when comparing prediabetic and diabetic subjects with healthy subjects, was for markers of insulin resistance. HOMA_IR_ values were markedly higher in prediabetic and diabetic subjects in both men (+75; +260%) and women (+112.0%; +306.0%) as compared to controls. With regard to insulin production, we only observed significant differences for diabetic subjects versus controls. Calculated HOMA_β_ values in prediabetic and diabetic men (−6.6%; −46.9%) and women (+0.3%; −37.9%) were most significantly reduced, versus controls, in diabetic subjects. In Figure 1, we have plotted HOMA_s_ (insulin sensitivity, the reciprocal of insulin resistance multiplied by 100) on the vertical axis versus insulin production (HOMA_β_) on the horizontal axis in all subjects. The data clearly indicate that while most diabetics are insulin resistant, a significant number also have evidence of decreased insulin production and relative insulin deficiency. Moreover, the median level of HOMA_β_ in diabetic subjects was about the same as the 25th percentile value in healthy subjects.

### 3.3. Comparison of Healthy, Prediabetic, and Diabetic Subjects—Inflammation

The most striking differences between prediabetic and diabetic subjects versus controls for inflammation markers, as shown in Table 1 and Table 2, were observed for hs-CRP, with much smaller differences for adiponectin, fibrinogen, myeloperoxidase (MPO), and especially for lipoprotein-associated phospholipase A_2_ (LpPLA_2_) (Table 2). Hs-CRP values were significantly higher in prediabetic and diabetic men (+20.0%; +90.0%) and women (+83.3%; +200.0%) versus controls. These differences for adiponectin in men were −8.5% and −20.2%, respectively, and in women −17.1% and −33.6%, respectively; and for fibrinogen in men +6.5% and +20.2% and in women +11.5% and +24.4%, versus controls. MPO values in prediabetic and diabetic men (+2.0%; +17.2%) and in women (+9.0%; +28.1%) were higher than in controls. For LpPLA_2_, the differences between prediabetic and diabetic men (−1.6%; −9.3%) and women (−1.6%; −9.7%) versus controls were quite modest.

The 10-year risk of diabetes, using the biochemical model developed from the Framingham Offspring Study, in healthy men was 0.6% and in women was 0.3%. For prediabetic men, this value was 11.6-fold higher at 7.0%, while for prediabetic women, it was 14-fold higher at 4.2% as compared to healthy subjects (Table 1).

### 3.4. Comparison of Healthy, Prediabetic, and Diabetic Subjects—Lipid Parameters

Table 3 shows that only very modest differences between prediabetic and diabetic subjects, versus controls, were observed for LDL-C, apolipoprotein (apo) B, and apoA-I, in contrast to fasting triglycerides, small dense low-density lipoprotein cholesterol (sdLDL-C), and HDL-C. Direct LDL-C values in prediabetic and diabetic men were −3.4% and −12.8%, respectively, and in women +5.4% and −2.6%, respectively, versus controls. For apoB, these differences in men were 0.0% and −2.1% and in women +5.4% and +7.5%, while the differences for apoA-I were in men −0.8% and −6.1% and in women −3.2% and −9.3%. In contrast, fasting triglyceride values in prediabetic and diabetic men (+12.6% and +44.7%, respectively) and women (+29.2% and +69.7%) were significantly higher than in controls, as were sdLDL-C values in men (+7.7; +19.2%) and women (+17.4%; +34.8) as compared to controls. HDL-C values in prediabetic and diabetic men were −6.1% and −18.4%, and in prediabetic and diabetic women, −10.9% and −23.4%, as compared to controls.

### 3.5. Effects of Lifestyle Modification in Prediabetes

In terms of the effects of lifestyle modification, as shown in Table 4, the biggest difference observed in prediabetic subjects was for predicted diabetes risk (−45.6% versus −1.6% in controls, *p* < 0.001), as determined by our model. Other significant effects of lifestyle change in this group were increases in adiponectin (+16.8% versus +10.7% in controls, *p* < 0.001) and decreases in triglycerides (−10.6% versus −6.3% in controls, *p* < 0.001), LDL-C (−11.1% versus 6.3% in controls, *p* < 0.011), and HOMA_IR_ (−11.8% versus −8.6% in controls, *p* = 0.023).

### 3.6. Effects of Lifestyle Modification in Diabetes

As compared with the control group, the effects of lifestyle modification were somewhat greater in diabetic subjects (Table 5) than in prediabetic subjects. The most important effect was that the percentage that were not diabetic was decreased by 8.2% in the lifestyle group versus 3.4% in the control group (*p* = 0.012), accompanied by weight reduction of 3.2% in the lifestyle group versus 0.9% in the control group (*p* = 0.004). Other significant effects were decreases in HbA1c (−5.6% versus −4.2% in controls, *p* = 0.031), GSP (−12.7% versus −8.0% in controls, *p* < 0.001), fasting glucose (−10.5% versus −7.8% in controls, *p* = 0.028), HOMA_IR_ (−29.5% versus −22.9% in controls, *p* = 0.048), hs-CRP (−30.8% versus −13.0% in controls, *p* < 0.001), LDL-C (−19.4% versus −7.1% in controls, *p* = 0.037), and fasting triglycerides (−15.6% versus −8.7%, *p* = 0.041). In both the prediabetic and the diabetic groups, the amount of weight loss in the lifestyle group correlated strongly with the amount of insulin resistance in both the prediabetic group (r = 0.675, *p* < 0.001) and in the diabetic group (r = 0.789, *p* < 0.001).

## 4. Discussion

It has long been known that subjects with diabetes are much more likely than healthy subjects to have obesity, elevated triglycerides, sdLDL-C, and hs-CRP levels, and decreased HDL-C levels, all important ASCVD risk factors [15,16,17,23,24]. Such differences are generally greater in older subjects versus younger subjects and more pronounced in women with diabetes than in men with diabetes. Moreover, since the development of insulin immunoassays, it has become clear that type 2 diabetes is often associated with both insulin resistance and decreased insulin production [25,26,27,28,29]. These findings have been facilitated by the development of equations for the calculation of HOMA_IR_ and HOMA_β_ [22,29]. In addition, the measurement of fasting insulin and the calculation of HOMA_IR_ and HOMA_β_ are essential for identifying subjects with diabetes requiring insulin therapy because of low insulin production, as seen in our population. Moreover, the most insulin-resistant subjects based on HOMA_IR_ values had the greatest weight loss, and the degree of weight loss (although modest) correlated well with the level of insulin resistance at baseline. In our view, it is in diabetic subjects that the measurement of insulin or C-peptide has the greatest value. Recently the measurement of C-peptide has been recommended in subjects that have diabetes for more than 3 years, and it is known that some patients develop a relative insulin deficiency for genetic reasons [30,31,32].

The efficacy of lifestyle modification in reducing the risk of developing diabetes by promoting exercise and weight loss has been shown to be in excess of 50% in prediabetic subjects in many studies, but generally very intensive counseling has been required [1,4,5,6]. In our large population study, our web-based personalized lifestyle program with a one-session review with a dietitian reduced diabetes risk in prediabetics by about 45% and in diabetics increased the remission rate from about 3% to about 8%. However, only 10–12% of subjects chose to voluntarily participate in this free program.

Digital health lifestyle interventions may be more practical than individual in-person visits with a dietitian. A recent meta-analysis on the effectiveness of digital interventions in prediabetics, with regard to glucose homeostasis and body weight, assessed 33 studies, consisting of 14,398 subjects, with study duration ranging from 3 to 60 months and study designs consisting of in-person meetings, telephone calls, or fully digital interventions. The investigators found that overall weight loss was −1.74 kg or 3.8 pounds in the intervention group, and some improvements in glucose homeostasis were seen. They concluded that digital health lifestyle interventions can result in a statistically significant change in body weight and other secondary outcomes among people with prediabetes [33].

In another recent meta-analysis, the effectiveness of different intervention modes—digital health, face-to-face, and blended interventions—in reducing diabetes and facilitating the reversion to non-diabetic status was assessed as compared to usual care [34]. The interventions demonstrated a significant 46% conversion to non-diabetic status compared to the usual care control group. Using only digital health interventions was associated with a 12% conversion to non-diabetic status. Interventions combining digital and face-to-face interventions were associated with an 87% increase in conversion to non-diabetic status. No significant effect on the reversal of prediabetes to normoglycemia was observed with digital health interventions. The investigators concluded that face-to-face interventions have consistently demonstrated promising effectiveness in reductions in diabetes incidence and in reversion to normoglycemia in adults with prediabetes, but this was not the case for digital interventions [34]. They recommended blended approaches, which is what we did, but only had one interaction between patients and dietitians.

Diabetes is a major risk factor for ASCVD [17]. The effectiveness of lifestyle interventions to reduce ASCVD risk was assessed in a meta-analysis of 29 studies consisting of 5490 adults with no CVD at baseline; 15 of these studies were randomized controlled trials (3605 subjects). The following lifestyle interventions were implemented: diet, physical activity, motivational interviewing, problem-solving, psychological counseling, cardiovascular risk assessment and feedback, health self-management education, and peer support. Various ASCVD risk assessment tools were used, including Framingham, SCORE, Heart Health Risk Assessment Score, Dundee, ASSIGN, and the UK Prospective Diabetes Study risk score. In the 15 randomized trials, lifestyle intervention reduced absolute ASCVD risk significantly [35]. The investigators concluded that lifestyle modification had a favorable impact on the absolute ASCVD risk score in adult populations without ASCVD at baseline.

Weight loss may not benefit all subjects with diabetes, especially those that have decreased insulin production, as more commonly seen in Asian populations. Such subjects may require insulin therapy [36,37]. Nevertheless, motivated individuals who follow a regular diet and exercise plan (daily 1 h exercise) can achieve and maintain significant weight loss and markedly improve their ASCVD risk [38]. Diet intervention can have a major impact on ASCVD risk reduction, especially if animal fat is substantially reduced along with sugar [39]. It has been clearly documented that compliance and follow-up with dietitians on a regular basis is key in lifestyle intervention weight loss programs [40]. However, many prediabetic and diabetic subjects require medications to achieve glycemic control [41,42]. More recently, glucagon-like peptide 1 receptor agonists (GLP-1RA) for type 2 diabetes and obesity have been developed and are now being widely used [43]. These medications have been shown to be very effective in promoting weight loss, diabetes reversal, and ASCVD risk reduction. They also may have efficacy for metabolic liver disease, peripheral artery disease, Parkinson disease, and Alzheimer’s disease. These medications can have significant side effects, and the long-term effects of these medications are not known. Moreover, once patients go off these medications, there is often substantial weight regain. Therefore, the cornerstone of therapy for prediabetes and diabetes remains lifestyle modification with diet and exercise.

It should be emphasized that the American Diabetes Association has stressed the importance of using fasting plasma glucose for diagnosing prediabetes and diabetes instead of fasting serum glucose [1]. Concern has been raised that serum values may be significantly lower than plasma values for glucose [44]. The fact that we used fasting serum glucose measurements may be a significant shortcoming and limitation of our studies. We have specified to our healthcare providers and phlebotomists the importance of the use of gel separator tubes, only allowing tubes to clot for 30 min, and centrifugation and placement of serum into transfer vials. In our own studies under such conditions, we have not noted significant differences between plasma and serum glucose values, and the Roche package insert also indicates that either serum or plasma can be used with their automated glucose assay. Nevertheless, this remains a potential significant limitation of our studies.

## 5. Conclusions

In conclusion, our studies are consistent with the following concepts: (1) diabetes risk prediction is useful in subjects with prediabetes, (2) a web-based personalized lifestyle program with professional review can be effective in reducing diabetes risk, and (3) the measurement of fasting glucose and insulin and the calculation of HOMA_IR_ and HOMA_β_ are useful for identifying diabetic subjects with insulin deficiency and low production who may require insulin therapy. The strength of our study is the large sample size we used. Potential weaknesses are that this was a retrospective analysis carried out only with information made available to the laboratory on the patient’s requisition and the fact that serum glucose was used instead of plasma glucose measurements.

## Figures and Tables

**Table 1:** Characteristics of prediabetic and diabetic subjects compared with healthy subjects.

Parameter	Healthy n = 75,271 (56.3%)		Prediabetic n = 48,455 (36.2%)		Diabetic n = 10,038 (7.5%)		% Difference, vs. Healthy Subjects
	N	Median (IQR)	N	Median (IQR)	N	Median (IQR)	
**Demographics**							
Age (years)	75,271	52.0 (22.0)	48,455	59.0 (17.0)	10,038	60.0 (17.0) *	+15.4%
Females	48,476	52.0 (22.0)	22,977	59.0 (17.0)	4175	60.0 (18.0) *	+15.4%
Males	26,795	52.0 (23.0)	25,478	58.0 (18.0)	5863	60.0 (17.0) *	+15.4%
Sex							
Females	48,476	64.4%	22,977	47.4%	4175	41.6% *	−35.4%
Males	26,795	35.6%	25,478	52.6%	5863	58.4% *	+64.0%
BMI (kg/m^2^)	12,794	27.0 (7.0)	11,758	30.0 (8.0)	801	32.0 (9.0) *	+18.5%
Females	8575	26.0 (8.0)	5605	30.0 (10.0)	331	34.0 (10.0) *	+30.8%
Males	4219	28.0 (7.0)	6153	30.0 (6.0)	470	31.0 (7.0) *	+10.7%
Weight (pounds)	34,298	169.0 (58.0)	22,078	191.0 (59.0)	4432	204.0 (65.0) *	+20.7%
Females	22,311	153.0 (49.0)	10,394	172.0 (57.0)	1850	188.0 (63.0) *	+22.9%
Males	11,987	195.0 (49.0)	11,684	205.0 (51.0)	2582	214.0 (60.0) *	+9.7%
**Metabolism**							
Glucose (mg/dL)	75,271	90.0 (10.0)	48,455	106.0 (10.0)	10,038	154.0 (57.0) *	+71.1%
Females	48,476	90.0 (9.0)	22,977	105.0 (9.0)	4175	153.0 (56.0) *	+70.0%
Males	26,795	92.0 (9.0)	25,478	106.0 (10.0)	5863	155.0 (57.0) *	+68.5%
Adiponectin (µg/mL)	75,271	12.6 (9.3)	48,455	10.1 (7.6)	10,038	8.3 (6.3) *	−34.1%
Females	48,476	14.6 (9.6)	22,977	12.1 (8.6)	4175	9.7 (7.4) *	−33.6%
Males	26,795	9.4 (6.4)	25,478	8.6 (5.9)	5863	7.5 (5.4) *	−20.2%
GSP (µmol/L)	75,269	199 (53)	48,454	205 (59)	10,038	299 (62) *	+50.3%
Females	48,476	198 (55)	22,976	200 (60)	4175	285 (98) *	+43.9%
Males	26,793	202 (52)	25,478	210 (58)	5863	308 (63) *	+52.5%
10 yr Diabetes Risk (%)	75,271	0.4 (0.6)	48,455	5.5 (12.1)	10,038	100.0	+250%
Females	48,476	0.3 (0.5)	22,977	4.2 (8.9)	4175	100.0	+333%
Males	26,795	0.6 (1.0)	25,478	7.0 (14.7)	5863	100.0	+167%

* *p* < 0.001 based on the non-parametric Kruskal–Wallis test. The top row is for all subjects. Percentage difference is between diabetic and healthy subjects.

**Table 2:** Characteristics of prediabetic and diabetic subjects compared with healthy subjects (metabolism and inflammation).

Parameter	Healthy n = 75,271 (56.3%)		Prediabetic n = 48,455 (36.2%)		Diabetic n = 10,038 (7.5%)		% Difference, vs. Healthy Subjects
	N	Median (IQR)	N	Median (IQR)	N	Median (IQR)	
**Metabolism**							
HbA1c (%)	72,980	5.5 (0.5)	45,176	5.7 (0.5)	9599	7.2 (1.9) *	+30.9%
Females	47,206	5.4 (0.5)	21,540	5.7 (0.5)	3989	7.2 (1.8) *	+33.3%
Males	25,774	5.5 (0.4)	23,636	5.7 (0.6)	5610	7.2 (1.9) *	+30.9%
Insulin (µU/mL)	73,624	8.0 (8.0)	45,176	13.0 (12.0)	9940	17.0 (18.0) *	+112.5%
Females	47,420	8.0 (7.0)	21,477	13.0 (12.0)	4130	18.0 (18.0) *	+125.0%
Males	26,204	9.0 (8.0)	23,699	13.0 (12.0)	5810	17.0 (19.0) *	+88.9%
HOMA_IR_	73,460	1.8 (1.8)	45,069	3.5 (3.3)	9781	7.3 (8.1) *	+305.6%
Females	47,331	1.7 (1.6)	21,421	3.6 (3.3)	4042	7.6 (8.0) *	+347.1%
Males	26,129	2.0 (2.0)	23,648	3.5 (3.3)	5739	7.2 (8.2) *	+260.0%
HOMA_β_	73,572	111 (102)	45,176	108 (95)	9940	64 (81) *	−42.1%
Females	47,390	109 (98)	21,477	110 (96)	4130	68 (83) *	−37.9%
Males	26,182	115 (111)	23,699	107 (96)	5810	62 (80) *	−46.2%
C-Peptide (ng/mL)	10,322	2.1 (1.3)	4897	3.2 (1.8)	1394	3.8 (2.4) *	+81.0%
Females	6597	2.0 (1.1)	2310	3.2 (1.8)	531	3.8 (2.5) *	+90.0%
Males	3725	2.3 (1.5)	2587	3.2 (1.8)	863	3.8 (2.3) *	+65.2%
**Inflammation**							
hs-CRP (mg/L)	72,717	1.1 (2.4)	45,569	1.6 (3.1)	9765	2.5 (4.4) *	+127.3%
Females	46,937	1.2 (2.7)	21,723	2.2 (4.1)	4052	3.6 (6.0) *	+200.0%
Males	25,780	1.0 (1.9)	23,846	1.2 (2.3)	5713	1.9 (3.3) *	+90.0%
Fibrinogen (mg/dL)	56,241	356 (108)	29,643	386 (113)	7621	433 (139) *	+21.6%
Females	36,635	356 (107)	13,976	397 (112)	3187	443 (132) *	+24.4%
Males	19,606	354 (108)	15,667	377 (113)	4434	425 (141) *	+20.2%
MPO (pmol/L)	58,752	263 (146)	34,592	274.0 (151)	8538	317 (180) *	+20.5%
Females	37,830	267 (149)	16,407	291 (160)	3547	342 (189) *	+28.2%
Males	20,922	256 (139)	18,185	261 (140)	4991	300 (170) *	+17.2%
LpPLA_2_ (nmol/min/mL)	71,783	184 (58)	44,587	180 (59)	9489	166 (61) *	−9.8%
Females	46,307	185 (57)	21,280	182 (60)	3946	167 (60) *	−9.7%
Males	25,476	182 (60)	23,307	179 (60)	5543	165 (63) *	−9.3%

* *p* < 0.001 based on the non-parametric Kruskal–Wallis test.

**Table 3:** Lipid and apolipoprotein concentrations of prediabetic and diabetic subjects compared with healthy subjects.

Parameter	Healthy n = 75,271 (56.3%)		Prediabetic n = 48,455 (36.2%)		Diabetic n = 10,038 (7.5%)		% Difference, vs. Healthy Subjects
	N	Median (IQR)	N	vs. Healthy Subjects	N	Median (IQR)	
LDL-C (mg/dL)	73,824	117.0 (51.0)	46,550	117.0 (54.0)	9966	107.0 (57.0) *	−8.5%
Females	47,539	117.0 (49.0)	22,127	121.0 (53.0)	4144	114.0 (59.0) *	−2.6%
Males	26,285	117.0 (54.0)	24,423	113.0 (55.0)	5822	102.0 (55.0) *	−12.8%
sdLDL-C (mg/dL)	73,572	24.0 (15.0)	46,258	27.0 (20.0)	9938	31.0 (25.0) *	+29.2%
Females	47,431	23.0 (14.0)	22,036	27.0 (18.0)	4135	31.0 (25.0) *	+34.8%
Males	26,141	26.0 (19.0)	24,222	28.0 (21.0)	5803	31.0 (25.0) *	+19.2%
HDL-C (mg/dL)	74,272	58.0 (24.0)	46,978	51.0 (22.0)	10,017	43.0 (17.0) *	−25.9%
Females	47,793	64.0 (25.0)	22,306	57.0 (22.0)	4161	49.0 (19.0) *	−23.4%
Males	26,479	49.0 (19.0)	24,672	46.0 (18.0)	5856	40.0 (15.0) *	−18.4%
Triglycerides (mg/dL)	75,271	93.0 (67.0)	48,455	116.0 (80.0)	10,038	150 (114) *	+61.3%
Females	48,476	89.0 (60.0)	22,977	115.0 (77.0)	4175	151 (107) *	+69.7%
Males	26,795	103.0 (75.0)	25,478	116.0 (83.0)	5863	149 (118) *	+44.7%
ApoA-I (mg/dL)	74,089	160.6 (44.4)	46,701	152.7 (40.6)	9994	142 (36.5) *	−11.8%
Females	47,705	170.5 (43.0)	22,166	165.0 (41.6)	4156	155 (38.8) *	−9.1%
Males	26,384	144.0 (34.7)	24,535	142.9 (33.8)	5838	135 (31.1) *	−6.3%
ApoB (mg/dL)	72,409	94.0 (36.0)	44,843	97.0 (38.0)	9715	96.0 (42.0) *	+2.1%
Females	46,616	93.0 (35.0)	21,338	98.0 (37.0)	4030	100.0 (44.0) *	+7.5%
Males	25,793	95.0 (38.0)	23,505	95.0 (39.0)	5685	93.0 (41.0) *	−2.1%

* *p* < 0.001 based on the non-parametric Kruskal–Wallis test.

**Table 4:** Effect of lifestyle intervention in prediabetic subjects.

Parameter *	Testing Only (N = 8559)					Testing and Life Plan (N = 1179)					*p* Value ^§^
	N	Baseline	Most Recent	% Change ^†^	*p* Value ^‡^	N	Baseline	Most Recent	% Change ^†^	*p* Value ^‡^	
Age (years)	61.0 (17.0)					60.0 (16.5)					
Females, %	3988 (46.6%)					646 (54.8%)					
Weight (pounds)	2988	190.0 (60.0)	188.0 (60.0)	−1.7%	0.004	569	189.0 (58.0)	187.0 (57.0)	−2.0%	<0.001	0.592
HbA1c (%)	7909	5.8 (0.5)	5.7 (0.5)	−0.0%	<0.001	1140	5.8 (0.5)	5.7 (0.5)	−0.1%	<0.001	<0.001
Glucose (mg/dL)	8559	106.0 (10.0)	104.0 (14.0)	−1.9%	<0.001	1179	107.0 (10.0)	104.0 (12.0)	−2.7%	<0.001	0.067
Insulin (µU/mL)	8098	13.0 (11.0)	12.0 (11.0)	−0.5%	<0.001	1150	13.0 (11.0)	12.0 (11.0)	−1.4%	<0.001	0.014
HOMA-IR	8081	3.5 (3.2)	3.2 (3.2)	−0.1%	0.004	1150	3.4 (3.1)	3.0 (3.0)	−0.4%	<0.001	0.023
HOMA-B	8095	106.4 (90.6)	110.0 (96.0)	+5.8%	<0.001	1150	102.9 (88.6)	105.4 (88.9)	+2.5%	0.542	0.252
hs-CRP (mg/L)	7941	1.5 (2.9)	1.4 (2.9)	−0.1%	0.145	1171	1.5 (3.0)	1.4 (2.7)	−0.2%	0.249	0.171
Adiponectin (µg/mL)	8559	9.8 (7.6)	10.7 (8.5)	+0.9%	<0.001	1179	9.5 (7.9)	11.1 (9.1)	+1.4%	<0.001	<0.001
GSP (µmol/L)	8559	211.0 (59.0)	202.0 (58.0)	−7.7%	<0.001	1179	207.0 (59.5)	199.0 (57.0)	−7.2%	<0.001	0.433
Fibrinogen (mg/dL)	5548	390.0 (109.2)	385.0 (113.0)	−2.6%	0.019	826	388.0 (110.0)	380.0 (109.0)	−7.6%	0.004	0.041
MPO (pmol/L)	5775	272.0 (144.5)	272.0 (150.0)	+2.2%	0.401	908	271.0 (149.0)	261.0 (144.2)	−11.0%	0.041	0.043
LpPLA_2_ nmol/min/mL	7758	179.0 (60.0)	172.0 (59.0)	−6.5%	<0.001	1110	183.0 (59.0)	170.5 (62.8)	−10.8%	<0.001	0.002
10 yr Diabetes Risk	8559	6.3 (14.1)	6.2 (12.1)	−1.6%	0.945	1179	5.7 (13.1)	3.1 (8.7)	−45.6%	<0.001	<0.001
LDL-C (mg/dL)	8157	112.0 (56.0)	105.0 (55.0)	−6.2%	<0.001	1178	117.0 (55.0)	107.0 (57.0)	−11.1%	<0.001	<0.001
sdLDL-C (mg/dL)	8071	27.0 (19.0)	25.0 (17.0)	−3.0%	<0.001	1176	28.0 (21.0)	24.0 (16.0)	−5.4%	<0.001	<0.001
HDL-C (mg/dL)	8316	51.0 (22.0)	51.0 (22.0)	+0.6%	<0.001	1178	52.0 (21.8)	53.0 (23.0)	+1.0%	<0.001	0.066
Triglycerides (mg/dL)	8559	116.0 (80.0)	109.0 (74.0)	−7.7%	<0.001	1179	113.0 (82.0)	101.0 (70.0)	−15.4%	<0.001	<0.001
ApoA-1 (mg/dL)	8286	153.9 (41.1)	152.2 (41.5)	−2.1%	<0.001	1172	157.1 (42.3)	153.0 (40.8)	−4.5%	<0.001	0.003
ApoB (mg/dL)	7895	94.0 (39.0)	90.0 (37.0)	−4.0%	<0.001	1120	97.0 (40.0)	89.0 (37.0)	−8.1%	<0.001	<0.001

* Parameters are expressed as median (IQR). ^†^ Percent change. ^‡^ *p*-value based on paired *t*-test, baseline compared with most recent test results. ^§^ *p*-value based on the effect of the Life Plan on the most recent test results after controlling for baseline values.

**Table 5:** Effect of lifestyle intervention in diabetic subjects.

Parameter	Testing Only (n = 2017)					Testing and Life Plan (n = 216)					*p* Value ^§^
	N	Baseline *	Most Recent *	% Change ^†^	*p* Value ^‡^	N	Baseline *	Most Recent *	% Change ^†^	*p* Value ^‡^	
Age (years)	63.0 (17.0)					62.0 (16.0)					
Females, %	773 (38.3%)					99 (45.8%)					
Weight (pounds)	719	205.0 (65.5)	203.0 (65.0)	−2.0%	<0.001	110	204.5 (65.0)	198.0 (60.5)	−6.2%	<0.001	0.004
HbA1c (%)	1867	7.2 (1.8)	6.9 (1.7)	−0.3%	<0.001	212	7.1 (1.5)	6.8 (1.4)	−0.5%	<0.001	0.001
Glucose (mg/dL)	2017	153.0 (54.0)	141.0 (57.0)	−17.1%	<0.001	216	152.0 (50.0)	136.5 (49.5)	−24.7%	<0.001	0.028
Insulin (µU/mL)	1997	17.0 (18.0)	15.0 (15.0)	−4.9%	<0.001	215	18.0 (19.0)	16.0 (13.0)	−4.3%	0.278	0.139
HOMA-IR	1980	7.0 (7.3)	5.4 (6.4)	−2.9%	<0.001	212	7.8 (8.2)	5.5 (5.6)	−1.1%	0.751	0.048
HOMA-B	1997	62.9 (79.1)	68.2 (83.4)	+4.3%	0.167	215	70.2 (78.1)	73.8 (76.3)	−7.4%	0.491	0.414
hs-CRP (mg/L)	1902	2.3 (4.1)	2.0 (3.8)	−0.5%	0.014	216	2.6 (4.1)	1.8 (3.4)	−0.5%	0.422	0.625
Adiponectin (µg/mL)	2017	8.0 (6.1)	9.0 (6.8)	+0.9%	<0.001	216	7.2 (5.0)	8.1 (6.0)	+1.1%	<0.001	0.425
GSP (µmol/L)	2017	301.0 (151.0)	277.0 (144.0)	−27.5%	<0.001	216	292.0 (127.8)	255.5 (110.5)	−50.6%	<0.001	<0.001
Fibrinogen (mg/dL)	1546	435.5 (142.8)	436.0 (134.0)	+2.8%	0.301	172	433.5 (128.8)	435.0 (129.0)	−0.5%	0.952	0.623
MPO (pmol/L)	1567	313.0 (174.0)	303.0 (170.5)	−6.6%	0.205	185	303.0 (192.0)	287.0 (178.0)	−20.9%	0.010	0.285
LpPLA_2_ nmol/min/mL	1840	168.0 (60.0)	157.0 (59.2)	−8.5%	<0.001	203	167.0 (64.5)	154.0 (59.0)	−14.6%	<0.001	0.076
Diabetes, %	2017	100.0	96.6	−3.4%	<0.001	216	100.0	91.8	−8.2%	<0.001	0.012
LDL-C (mg/dL)	2000	103.0 (59.0)	94.0 (53.0)	−7.1%	<0.001	216	108.0 (55.8)	87.0 (56.5)	−12.4%	<0.001	0.037
sdLDL-C (mg/dL)	1989	31.0 (26.0)	26.0 (21.0)	−4.3%	<0.001	216	32.0 (27.2)	24.0 (18.0)	−8.0%	<0.001	0.005
HDL-C (mg/dL)	2016	43.0 (17.0)	44.0 (18.0)	+0.6%	0.001	216	43.0 (15.5)	43.5 (18.2)	+0.9%	0.110	0.755
Triglycerides (mg/dL)	2017	150.0 (108.0)	137.0 (100.0)	−16.4%	<0.001	216	154.0 (114.2)	130.5 (88.0)	−30.7%	0.004	0.174
ApoA-I (mg/dL)	2007	143.0 (37.3)	141.0 (36.9)	−2.7%	<0.001	216	144.3 (36.1)	140.5 (34.1)	−6.3%	<0.001	0.008
ApoB (mg/dL)	1984	93.0 (43.0)	87.0 (38.0)	−5.2%	<0.001	212	96.0 (42.0)	81.5 (35.2)	−11.5%	<0.001	0.004

* Parameters are expressed as median (IQR). ^†^ Percent change. ^‡^ *p*-value based on paired *t*-test, baseline compared with most recent test results. **^§^** *p*-value based on the effect of the Life Plan on the most recent test results after controlling for baseline values.

## Data Availability

The data presented in this study may be available on request from the corresponding authors. Restrictions may apply due to legal reasons.

## Abbreviations

ASCVD	atherosclerotic cardiovascular disease
apo	apolipoprotein
BMI	body mass index
GLP-1RA	glucagon-like peptide 1 receptor agonist
GSP	glycated serum protein
HbA1c	glycosylated hemoglobin
HDL-C	high-density lipoprotein cholesterol
HOMA_β_	homeostasis model of insulin production
HOMA_IR_	homeostasis model of insulin resistance
HOMA_s_	homeostasis model of insulin sensitivity
hs-CRP	high sensitivity C-reactive protein
LDL-C	low-density lipoprotein cholesterol
LpPLA_2_	lipoprotein-associated phospholipase A_2_
MPO	myeloperoxidase
sdLDL-C	small dense low-density lipoprotein cholesterol
